# Analysis of the ATR-Chk1 and ATM-Chk2 pathways in male breast cancer revealed the prognostic significance of ATR expression

**DOI:** 10.1038/s41598-017-07366-7

**Published:** 2017-08-14

**Authors:** Anna Di Benedetto, Cristiana Ercolani, Marcella Mottolese, Francesca Sperati, Laura Pizzuti, Patrizia Vici, Irene Terrenato, Abeer M. Shaaban, Matthew P. Humphries, Luigi Di Lauro, Maddalena Barba, Ilio Vitale, Gennaro Ciliberto, Valerie Speirs, Ruggero De Maria, Marcello Maugeri-Saccà

**Affiliations:** 1 Department of Pathology, “Regina Elena” National Cancer Institute, Via Elio Chianesi 53, 00144 Rome, Italy; 2 Biostatistics-Scientific Directorate, “Regina Elena” National Cancer Institute, Via Elio Chianesi 53, 00144 Rome, Italy; 3 Division of Medical Oncology 2, “Regina Elena” National Cancer Institute, Via Elio Chianesi 53, 00144 Rome, Italy; 4 Queen Elizabeth Hospital Birmingham and University of Birmingham, Department of Histopathology, Edgbaston, Birmingham B15 2GW UK; 5 Leeds Institute of Cancer and Pathology, Wellcome Trust Brenner Building, University of Leeds, Leeds, LS9 7TF UK; 6 Scientific Directorate, “Regina Elena” National Cancer Institute, Via Elio Chianesi 53, 00144 Rome, Italy; 7 Department of Biology, University of Rome “Tor Vergata”, Via della Ricerca Scientifica 1, 00133 Rome, Italy; 8 Institute of General Pathology, Catholic University of the Sacred Heart, Largo Agostino Gemelli, 10, 00168 Rome, Italy

## Abstract

The ATR-Chk1 and ATM-Chk2 pathways are central in DNA damage repair (DDR) and their over-activation may confer aggressive molecular features, being an adaptive response to endogenous DNA damage and oncogene-induced replication stress. Herein we investigated the ATR-Chk1 and ATM-Chk2 signalings in male breast cancer (MBC). The expression of DDR kinases (pATR, pATM, pChk1, pChk2, and pWee1) and DNA damage markers (pRPA32 and γ-H2AX) was evaluated by immunohistochemistry in 289 MBC samples to assess their association. Survival analyses were carried out in 112 patients. Survival curves were estimated with the Kaplan-Meier method and compared by log-rank test. Cox proportional regression models were generated to identify variables impacting survival outcomes. The expression of pATR conferred poorer survival outcomes (log rank p = 0.013, p = 0.007 and p = 0.010 for overall, 15- and 10-year survival, respectively). Multivariate Cox models of 10-year survival and overall indicated that pATR expression, alone or combined with pChk2, was an independent predictor of adverse outcomes (10-year survival: pATR: HR 2.74, 95% CI: 1.23–6.10; pATR/pChk2: HR 2.92, 95% CI: 1.35–6.33; overall survival: pATR: HR 2.58, 95% CI: 1.20–5.53; pATR/pChk2: HR 2.89, 95% CI: 1.37–6.12). Overall, the ATR/ATM-initiated molecular cascade seems to be active in a fraction of MBC patients and may represent a negative prognostic factor.

## Introduction

Male breast cancer (MBC) is a rare neoplasm that shares similarities with post-menopausal breast cancer^1, 2^. Indeed, MBC is a disease of elderly men, which frequently expresses steroid receptors, namely the estrogen receptor (ER), progesterone receptor (PgR) and androgen receptor (AR)^1, 3^. The hormone-dependent nature of MBC is exploited for therapeutic purposes, since hormone therapies including tamoxifen^4^, aromatase inhibitors^5–7^, fulvestrant^8^, GnRH analogues^9^, and antiandrogens^10^ have shown antitumor activity, albeit in retrospective studies.

Impressive advancements have been made in the molecular characterization of tumours over the last decade. As a result, most common tumors were stratified into a number of subtypes, each characterized by specific genomic alterations and deregulated pathways. Although the rarity of MBC has hindered comprehensive characterization efforts, initial clues on the nature of its genetic abnormalities are beginning to be elucidated^11–17^. Recently, massive parallel sequencing of 241 genes frequently mutated in female breast cancer has been applied to a series of 59 MBC samples, reporting a significant enrichment for mutations/copy number variations in DNA repair–related genes^17^. Results suggest that, in MBC, the DNA damage response (DDR) machinery is targeted by genetic abnormalities at multiple levels, including central players of the apoptotic response (e.g. *TP53*), effectors/mediators of the DDR (e.g. *CHEK1*, *PALB2*, and *BRCA2*), and mechanisms fueling oncogene-induced replication stress (e.g. *MYC* amplification). In this small-sized case series, a suggestion toward inferior survival outcomes was observed in patients whose tumors harbored DDR alterations.

The DNA damage response (DDR) is a sophisticated molecular network deputed to maintain genomic stability by correcting DNA damage or eliminating cells whose damage overwhelms repair capabilities^18^. The DDR encompasses a number of pathways that are activated by the presence of single-stranded DNA (ssDNA), DNA single- and double-strand breaks (SSBs and DSBs, respectively). Schematically, DDR pathways can be grouped into: (i) cell cycle checkpoints that halt the progression of the cell cycle, (ii) DNA repair mechanisms that remove DNA lesions, (iii) DNA damage tolerance processes that enable cells to withstand persisting lesions in the absence of repair, and (iv) cell death pathways that eliminate irremediably damaged cells^19^.

The ataxia telangiectasia mutated (ATM) kinase is activated upon the onset of DSBs, whereas the ataxia telangiectasia and Rad3-related protein (ATR) recognizes ssDNA and SSBs^20, 21^. Upon their recruitment to DNA damage sites, ATM and ATR activate the Checkpoint Kinase 2 (Chk2) and Checkpoint kinase 1 (Chk1), respectively, even though an extensive communication exists between the two signaling avenues. Overall, the AMT-Chk2 and ATR-Chk1 pathways, together with the Wee1-like protein kinase (Wee1) that is activated by Chk1, are crucial for determining cell fate upon genotoxic injuries, being central in the G_1_-S, intra-S and G_2_-M cell cycle checkpoints^20, 21^.

Our previous results, both at the preclinical and clinical level, suggest that abnormal DDR activation is linked to suboptimal efficacy of chemotherapy^22–25^. Herein we hypothesized that over-activation of ATM-ATR-initiated signaling may configure a subset of MBC endowed with more aggressive molecular traits, assuming that this process reflects an underlying genetic portrait dominated by deregulated cell cycle control systems (e.g. *TP53* mutations) and elevated levels of oncogene-induced replication stress (e.g. *MYC* amplification)^26, 27^. To test this hypothesis, a large series of MBC samples were immunostained for evaluating the expression of central DDR kinases, namely phosphorylated (activated) ATR (pATR), ATM (pATM), Chk1 (pChk1), Chk2 (pChk2), and Wee1 (pWee1). The panel of candidate biomarkers was completed by the assessment of the DNA DSB marker phosphorylated H2A Histone Family Member X (γ-H2AX) and the replication stress marker phosphorylated replication protein A2 (pRPA2, best known as pRPA32).

## Results

### Characteristics of the patients and expression pattern of DDR biomarkers

**Figure 1:**
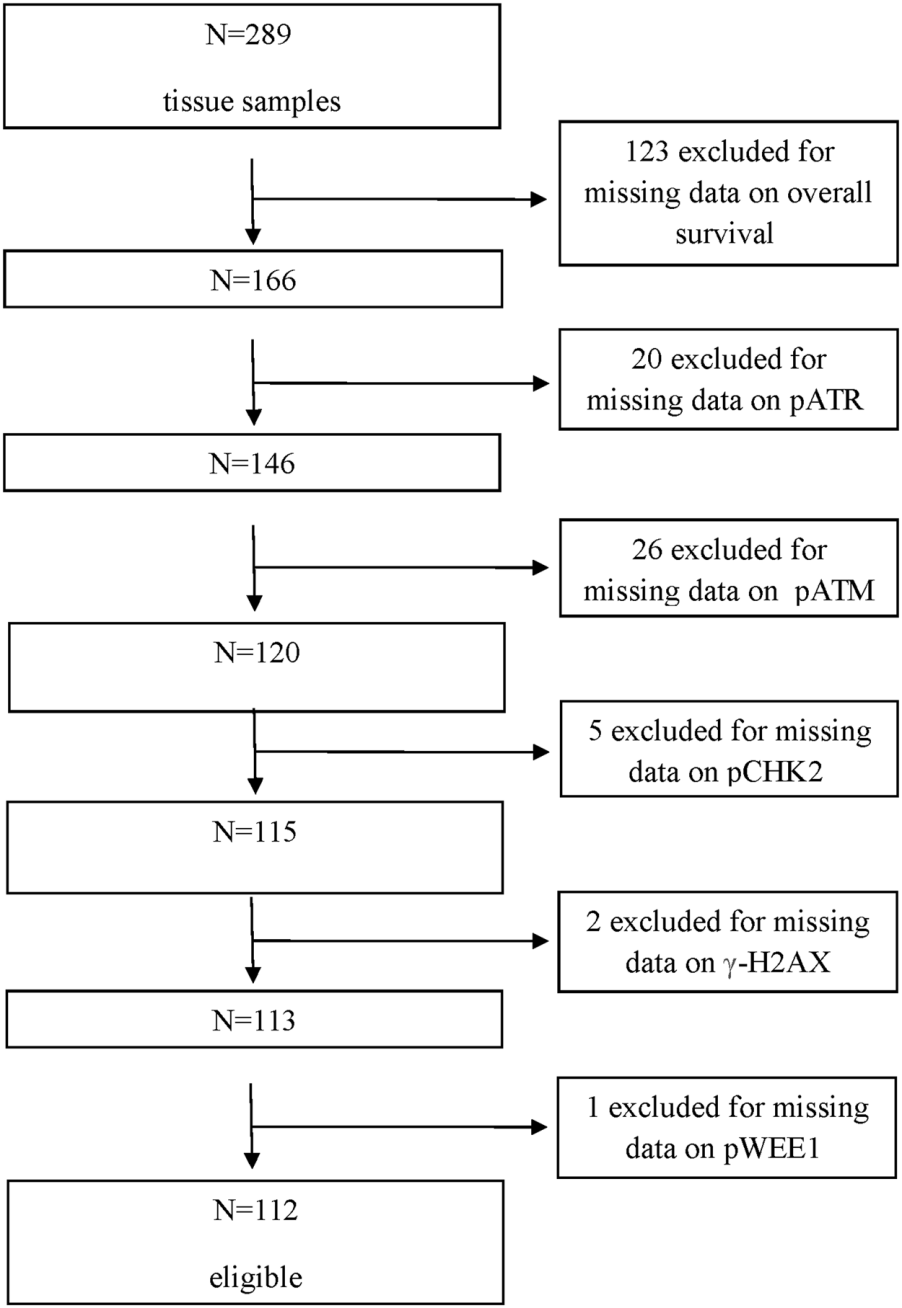
Flow diagram of the patients’ selection process.

**Figure 2:**
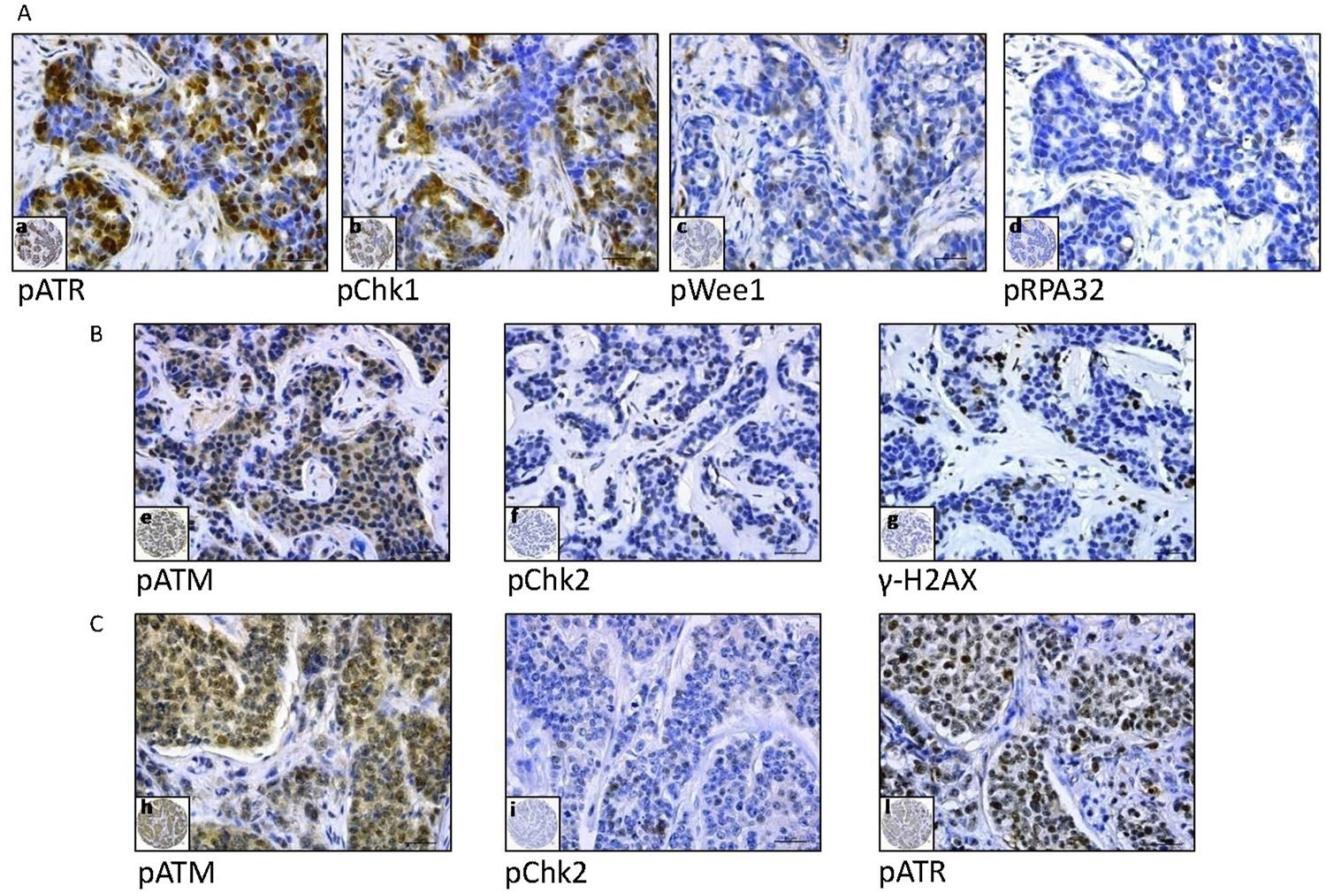
Representative examples of immunohistochemical expression of pATR, pChk1, pWee1, pRPA32, pATM, pChk2 and γ-H2AX in three male breast cancer patients (**A**,**B**,**C**). Panels a–d show a sample with nuclear expression of pATR, pCHK1, pWEE1 and pRPA32 (**A**). Panels e–g show a sample with nuclear expression of pATM, pCHK2 and γ-H2AX (**B**). Panels h–l show a sample with nuclear expression of pATM, pCHK2 and pATR (**C**). Slide magnification ×40, inset magnification ×10. Scale bar 30 μm.

**Figure 3:**
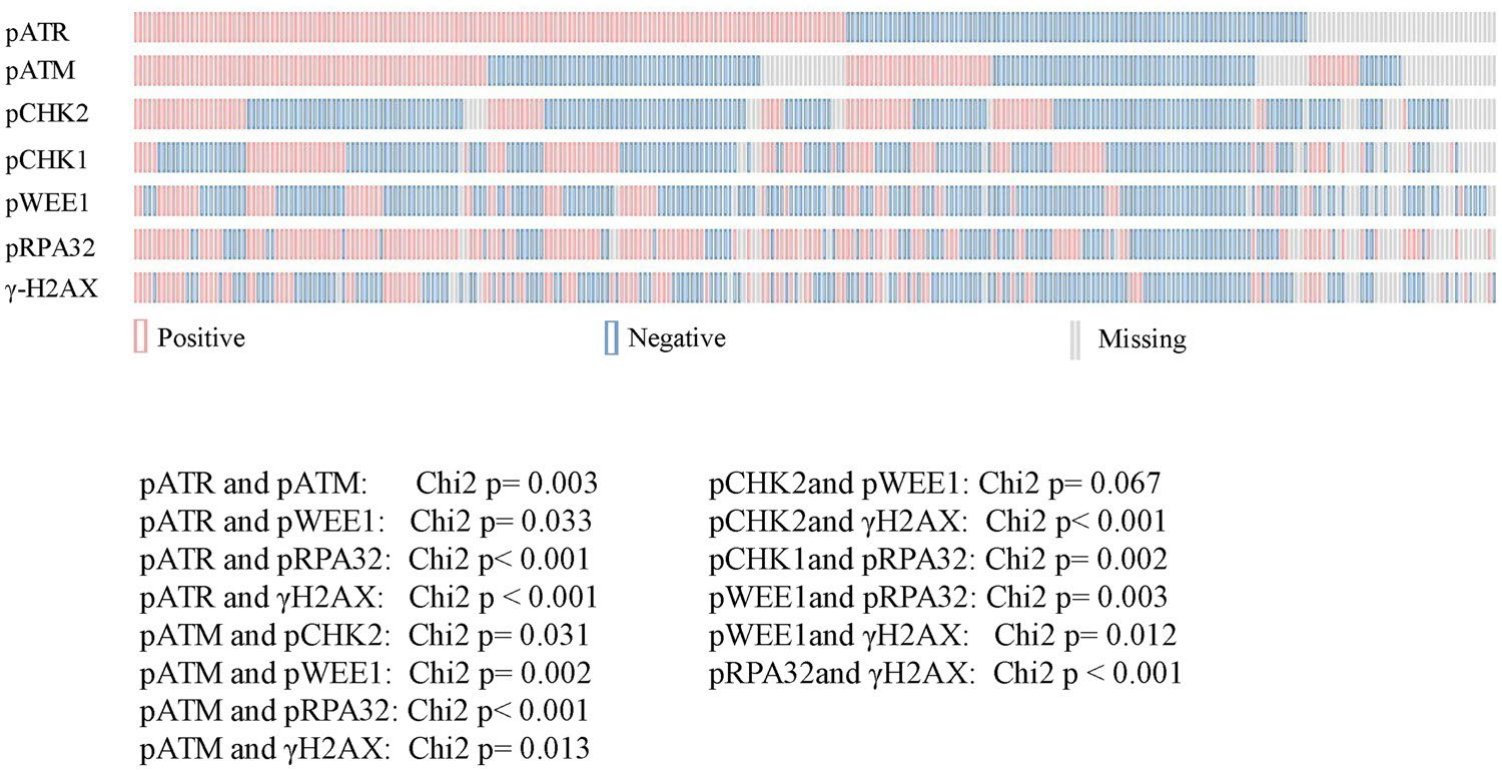
OncoPrint showing the associations between DDR-related biomarkers in male breast cancer. Statistically significant or borderline significant associations are reported.

Baseline characteristics of MBC patients whose biological samples were immunostained (N = 289) are summarized in Table 1, together with the characteristics of patients who met the eligibility criteria for the analysis of survival outcomes (N = 112). The flow diagram illustrating patients’ selection process is detailed in Fig. 1. Representative immunohistochemical staining is presented in Fig. 2. We first investigated whether the expression pattern of DDR biomarkers was consistent with the hypothesis of activated ATR-ATM signaling in MBC. As presented in Fig. 3, a significant association (co-expression) was observed between many of the investigated markers. Further enforcing our hypothesis, we recorded multiple significant correlations when DDR markers were analyzed as continuous variables (percentage of nuclear-expressing tumor cells), as presented in Supplementary Table 1. When evaluating the relationship between DDR markers and standard clinical, pathological and molecular features (detailed in Table 1), the only significant associations were between pChk1 and age (Chi2 p = 0.046) and pATR and age (Chi2 p = 0.048). Indeed, both biomarkers were more expressed in tumors from older patients (data available upon request).

**Table 1:** Baseline characteristics of MBC patients included in this study.

Characteristics		Overall population (N = 289)	Population included in survival analyses (N = 112)
Age at diagnosis	Median (min-max) [IQ range]	66.8 (30–97) [58–76]	66.5 (34–89) [58.5–75]
	Not Available	54	6
Histology N(%)	IDC/ILC	218 (75.4)	92 (82.1)
	Other	53 (19.6)^*^	20 (17.9)^**^
	Not Available	17 (5.0)	—
Grade N(%)	G1–2	149 (51.6)	65 (58.0)
	G3	115 (39.8)	47 (42.0)
	Not Available	25 (8.6)	—
Nodal status N(%)	Negative	93 (32.2)	39 (34.8)
	Positive	105 (36.3)	52 (46.4)
	Not Available	91 (31.5)	21 (18.8)
Hormone Receptors N(%)	ER^+^/PgR^+^	221 (76.5)	91 (81.2)
	Other	37 (12.8)	21 (18.8)
	Not Available	31 (10.7)	—
Ki-67 N(%)	Low (<14%)	116 (40.1)	55 (49.1)
	High (≥14%)	67 (23.2)	44 (39.3)
	Not Available	106 (36.7)	13 (11.6)

*10 Adenocarcinoma, 5 Intraductal papillary carcinoma, 10 Papillary carcinoma, 11 Mucinous carcinoma, 5 Ductal carcinoma *in situ*, 6 Mixed, 1 Medullary, 2 Micropapillary, 2 Cribiform, 1 Tubular.

**3 Adenocarcinoma, 4 Intraductal papillary carcinoma, 6 Papillary carcinoma, 1 Mucinous carcinoma, 1 Mixed, 1 Medullary, 2 Micropapillary, 1 Cribiform, 1 Tubular.

Abbreviations: IDC: Invasive Ductal Carcinoma, ILC: Invasive Lobular Carcinoma.

### DDR biomarkers and survival outcomes

**Figure 4:**
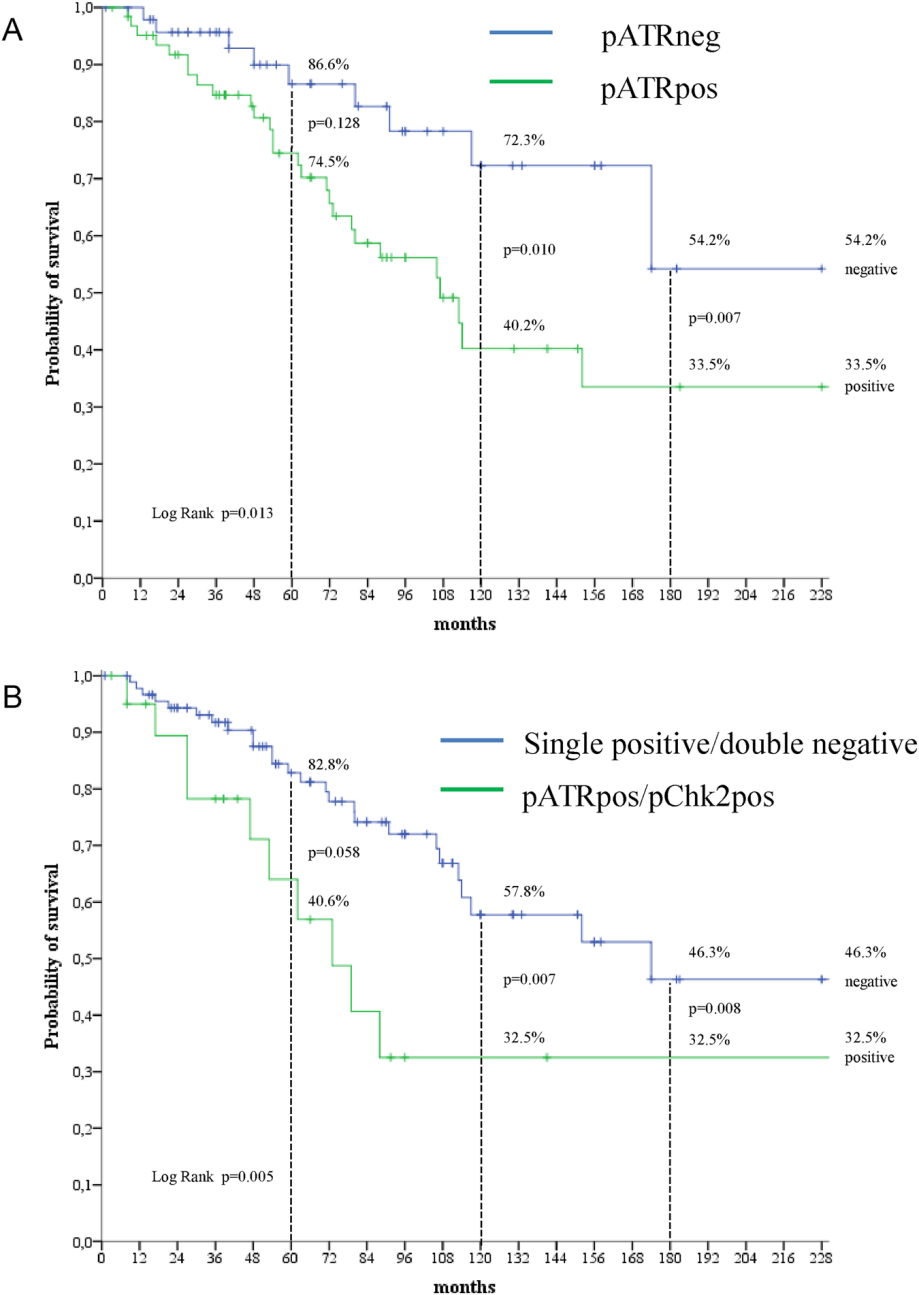
Kaplan-Meier survival curves comparing (**A**) pATR-positive and pATR-negative cases, and (**B**) pATR/pChk2-positive and single-positive/double-negative tumors (N = 112).

Patients whose tumors expressed pATR (N = 63/112, 56.3%) had poorer survival outcomes compared with their negative counterparts (log rank p = 0.013, p = 0.007 and p = 0.010 for OS, 15- and 10-year survival, respectively, Fig. 4 panel A). The combined expression of pATR and pChk2 (N = 21/112, 18.8%) was associated with similar adverse survival outcomes than pATR alone, with a trend toward statistical significance for 5-year survival (log rank p = 0.058, Fig. 4 panel B). A model of triple-positivity (pATR/pChk2/pATM, N = 10/112, 8.9%) was fully significant also for 5-year survival (log rank p = 0.048), although the number of patients falling into this molecular group is limited (data available upon request).

Univariate Cox regression analyses (Table 2) carried out for identifying factors impacting 10-year survival confirmed the adverse prognostic significance of pATR, pATR/pChk2 and pATR/pChk2/pATM (pATR: HR 2.71, 95% CI: 1.22–5.99, p = 0.014; pATR/pChk2: HR 2.67, 95% CI: 1.27–5.62, p = 0.010; pATR/pChk2/pATM: HR 3.76, 95% CI: 1.69–8.35, p = 0.001). Multivariate Cox regression models (Table 2) generated by adjusting for other plausible predictors of the outcome of interest confirmed these results (pATR: HR 2.74, 95% CI: 1.23–6.10, p = 0.014; pATR/pChk2: HR 2.92, 95% CI: 1.35–6.33, p = 0.007; pATR/pChk2/pATM: HR 4.76, 95% CI: 2.05–11.04, p < 0.001). As shown in Table 3, uni- and multivariate Cox regression models for OS yielded comparable results (pATR multivariate Cox: HR 2.58, 95% CI: 1.20–5.53, p = 0.015; pATR/pChk2 multivariate Cox: HR 2.89, 95% CI: 1.37–6.12, p = 0.005; pATR/pChk2/pATM multivariate Cox: HR 4.71, 95% CI: 2.04–10.86, p < 0.001). A sensitivity analysis carried out on a subset of 99 patients with available data on Ki-67 that considered Ki-67 among the variables of interest, confirmed the adverse impact of DDR markers on 10-year survival (pATR multivariate Cox: HR 2.65, 95% CI:1.15–6.12, p = 0.023; pATR/pChk2 multivariate Cox: HR: 3.24, 95% CI: 1.40–7.47, p = 0.006; pATR/pChk2/pATM multivariate Cox: HR 5.16, 95% CI: 2.07–12.83, p < 0.001) (Table 4). Finally, in subgroup analysis, pATR expression and the pATR/pChk2 co-expression model were associated with an increased risk of death within a 10-year timeframe in the subgroup of patients with elevated Ki-67 levels and with node-positive disease (Supplementary Figure 1), although to a borderline significant extent. In these subgroups, the pATR/pChk2/pATM model was fully significant in the subset of patients with elevated Ki-67 levels in their tumors and with nodal involvement (HR 7.44, 95% CI: 2.18–25.46, p = 0.001; and HR 9.66, 95% CI 2.75–33.87, p < 0.001, respectively; data available upon request).

**Table 2:** Univariate and multivariate Cox regression models for 10-year survival (N = 112).

		Univariate Cox regression model		Multivariate Cox regression model^§^		Multivariate Cox regression model^§^		Multivariate Cox regression model^§^	
		HR (95% CI)	p-value	HR (95% CI)	p-value	HR (95% CI)	p-value	HR (95% CI)	p-value
Histology	IDC/ILC vs other	1.32 (0.46–3.75)	0.602	1.05 (0.36–3.06)	0.922	1.41 (0.48–4.13)	0.530	1.52 (0.52–4.43)	0.448
Grade	G3 vs G1-2	1.84 (0.94–3.61)	0.077	1.78 (0.90–3.55)	0.098	1.69 (0.85–3.36)	0.135	2.02 (1.00–4.07)	0.050
Hormone Receptors	ER^+^/PgR^+^ vs other	0.79 (0.36–1.76)	0.567	0.75 (0.34–1.68)	0.487	0.68 (0.30–1.56)	0.365	0.76 (0.33–1.71)	0.504
pATR	Pos vs Neg	2.71 (1.22–5.99)	0.014	2.74 (1.23–6.10)	0.014	—	—	—	—
pATR/pChk2	Pos vs Neg	2.67 (1.27–5.62)	0.010	—	—	2.92 (1.35–6.33)	0.007	—	—
pATR/pChk2/pATM	Pos vs Neg	3.76 (1.69–8.35)	0.001	—	—			4.76 (2.05–11.04)	<0.001

^§^ **Adjusted for**: Histology, Grade, Hormone Receptor Status.

**Table 3:** Univariate and multivariate Cox regression models for overall survival (N = 112).

		Univariate Cox regression model		Multivariate Cox regression model^§^		Multivariate Cox regression model^§^		Multivariate Cox regression model^§^	
		HR (95% CI)	p-value	HR (95% CI)	p-value	HR (95% CI)	p-value	HR (95% CI)	p-value
Histology	IDC/ILC vs other	1.32 (0.46–3.75)	0.602	1.07 (0.37–3.09)	0.904	1.44 (0.49–4.22)	0.503	1.52 (0.52–4.42)	0.445
Grade	G3 vs G1-2	1.74 (0.92–3.32)	0.092	1.74 (0.90–3.35)	0.100	1.54 (0.80–2.98)	0.200	1.90 (0.97–3.69)	0.059
Hormone Receptors	ER^+^/PgR^+^ vs other	0.79 (0.37–1.68)	0.538	0.74 (0.35–1.59)	0.446	0.65 (0.29–1.43)	0.283	0.74 (0.34–1.59)	0.435
pATR	Pos vs Neg	2.52 (1.18–5.37)	0.017	2.58 (1.20–5.53)	0.015	—	—	—	—
pATR/pChk2	Pos vs Neg	2.67 (1.31–5.46)	0.007	—	—	2.89 (1.37–6.12)	0.005	—	—
pATR/pChk2/pATM	Pos vs Neg	3.76 (1.69–8.35)	0.001	—	—	—	—	4.71 (2.04–10.86)	<0.001

^§^ **Adjusted for**: Histology, Grade, Hormone Receptor Status.

**Table 4:** Univariate and multivariate Cox regression models for 10-year survival in patients with available information on Ki-67 levels (N = 99).

		Univariate Cox regression model		Multivariate Cox regression model^§^		Multivariate Cox regression model^§^		Multivariate Cox regression model^§^	
		HR (95% CI)	p-value	HR (95% CI)	p-value	HR (95% CI)	p-value	HR (95% CI)	p-value
Histology	IDC/ILC vs other	1.16 (0.40–3.43)	0.785	1.06 (0.35–3.18)	0.919	1.29 (0.43–3.85)	0.644	1.41 (0.47–4.20)	0.541
Grade	G3 vs G1-2	1.81 (0.86–3.81)	0.119	1.63 (0.75–3.54)	0.219	1.52 (0.69–3.34)	0.302	1.77 (0.80–3.91)	0.161
Hormone Receptors	ER^+^/PgR^+^ vs other	0.65 (0.27–1.53)	0.332	0.58 (0.24–1.40)	0.224	0.54 (0.22–1.34)	0.185	0.65 (0.27–1.58)	0.342
Ki67	High vs Low	1.24 (0.59–2.60)	0.575	1.27 (0.59–2.72)	0.537	1.13 (0.53–2.43)	0.748	1.29 (0.59–2.81)	0.529
pATR	Pos vs Neg	2.45 (1.08–5.57)	0.033	2.65 (1.15–6.12)	0.023	—	—	—	—
pATR/pChk2	Pos vs Neg	2.91 (1.31–6.45)	0.009	—	—	3.24 (1.40–7.47)	0.006	—	—
pATR/pChk2/pATM	Pos vs Neg	4.08 (1.73–9.62)	0.001	—	—	—	—	5.16 (2.07–12.83)	<0.001

^§^ **Adjusted for**: Histology, Grade, Hormone Receptor Status, Ki67.

## Discussion

In the present study, we investigated whether core components of the DDR are involved in MBC, and whether this process translates into adverse survival outcomes. Overall, our pathway-level analysis suggests that a portion of MBC carries activation of the molecular cascade safeguarding the genome and the transmission of an unaltered genetic code to daughter cells. Furthermore, we identified elevated pATR expression, both alone or with pChk2 and eventually in a triple model that considers pATM, as a plausible negative prognostic factor for MBC patients.

The present collection of MBC samples was already exploited for translational studies investigating stem cell and metabolic pathways^28–30^. As discussed earlier, the rarity of the disease and the correlated need for collecting samples across multiple countries and in a wide time window (1983–2009) resulted in the partial unavailability of some information. The unestablished cause of death for all the patients analyzed (37 deaths, 13 cancer-related deaths, 24 deaths from unknown cause), together with fragmented data on adjuvant therapy, need to be carefully considered. Aware of these intrinsic limitations, we analyzed the impact of DDR markers on survival outcomes at different time-points, deducing that this approach may soften the partial unavailability of cancer-specific events, especially in consideration of the fact that an elderly patient’ population was the focus of the present investigation. Regarding adjuvant therapy, the data gathered indicate a therapeutic approach that is consistent with current recommendations. Indeed, tamoxifen is the therapy of choice in the adjuvant setting, despite the high discontinuation rate in MBC patients (e.g. sexual dysfunction and thromboembolic events) legitimates the use of aromatase inhibitors^31^.

Nevertheless, our study offers some intriguing clues on the functional status of a commonly deregulated pathway in cancer. First, the co-expression pattern observed for many biomarkers indicates activation of upstream branches of the DDR in a subset of MBC, a process supposedly correlated with the need for compensating genetic defects that deregulate, and overall accelerate, the transition between the various phases of the cell cycle and that hinder the apoptotic response. Second, our data point to the machinery that senses the presence of ssDNA and SSBs as an adverse prognostic factor for these patients, plausibly mirroring the replication stress imposed by the constitutive activation of oncogene involved in cell proliferation^26^. Interestingly, the negative prognostic significance of pATR expression was enhanced by the concomitant expression of pChk2 (and also pATM), suggesting a communication between the ATR-Chk1 and ATM-Chk2 pathways. Historically, these signals were thought to act in parallel with partly overlapping functions. This view changed over time, and now we know that they have non-redundant functions in the DDR cascade^32^. Third, ATM/ATR-initiated signal is required for an array of repair pathways, spanning from non-homologous end joining (NHEJ), homologous recombination, nucleotide excision repair, interstrand crosslink (ICL) repair and replication fork stability^33^. Consistently with the complexity of the molecular network initiated by ATR and ATM, hundreds of proteins are phosphorylated in an ATM/ATR-dependent manner^34^. In consideration of the complexity of the DDR, we decided to examine core pathway components acting upstream the DDR cascade and coordinating the cellular response upon DNA damage. Importantly, the DDR kinases herein evaluated are potentially targetable with compounds that have already entered early phases of clinical development^35, 36^, and the significance of their expression may be a molecular trait MBC shares with female breast cancer^37, 38^.

The long-term strategy we envision for streamlining the development of DDR-linked biomarkers relies on a more exhaustive characterization of the pathway, which takes into account the origin of DNA damage in cancer. The genome of eukaryotic cells is susceptible to a variety of endogenous and exogenous cues, and this process is greatly intensified upon malignant transformation. A number of intertwined factors account for this including: (i) changes in cellular bioenergetics (metabolic reprogramming) that lead to an elevated production of byproducts, such as reactive oxygen species, capable of damaging the DNA^39^, (ii) activating mutations in oncogenes that accelerate cellular proliferation consequently resulting in replication stress (stalled or collapsed replication forks)^26^, and (iii) loss-of-function mutations in genes that restrain cellular proliferation^27^. All these conditions require a compensatory response to avoid that damaged cells embark into a defective, potentially catastrophic, mitosis. Over-activation of the ATM-Chk2 and ATR-Chk1 pathways was identified as a strategy neoplastic cells exploit to counteract the excess of DNA-damaging insults they are exposed. Consistently, the pharmacological targeting of DDR kinases was conceived as a synthetically lethal therapeutic approach for tumors carrying a defective G1-S transition machinery (*TP53* defects) or characterized by elevated levels of replication stress (*MYC* amplification)^40, 41^. On this premise, we hypothesize that a deeper characterization of the DDR, envisioning the simultaneous assessment of the ATM-ATR cascade together with mutations/copy number variations in genes that, at the protein level, participate in the DDR response may accelerate the development of DDR biomarkers. For instance, BRCA2 mutations or rearrangements and, to a lower extent, BRCA1 mutations have been described in MBC^42^. *BRCA1* and *BRCA2* encode for two proteins that are central in the repair of DSBs by homologous recombination, being therefore intimately tied to ATR and ATM^36^. Remarkably, beyond the established sensitivity of BRCA1/2-mutated tumors to inhibitors of poly(ADP-ribose)-polymerase (PARP), BRCA1- deficient cells were found to be sensitive to ATM inhibition^43^. Collectively, a series of evidence highlight the potential of DDR-targeting agents for the treatment of MBC patients.

Overall, our results suggest that the molecular network that senses, signals and amplifies the presence of genetic lesions is active in MBC, and patients whose tumors display this molecular portrait may have adverse survival outcomes. A larger characterization of the DDR is warranted to obtain more accurate information on how deregulation of this complex molecular network affects the prognosis, and in future perspective treatment, of MBC patients.

## Material and Methods

### Study Participants

Tissue microarrays (TMAs) including samples from 289 histologically confirmed MBC patients were immunohistochemically characterized for evaluating the expression of five DDR kinases (pATR, pATM, pChk1, pChk2 and pWee1) and two DNA damage markers (pRPA32 and γ-H2AX). The relationship between the biomarkers of interest was assessed in the entire cohort (N = 289), whereas their prognostic significance was evaluated in 112 patients based on the following criteria: (i) available information on survival outcomes, and (ii) complete data pertinent to the investigated markers. Given that data related to Ki-67 levels and nodal status were available for 99 and 91 patients, respectively, these subsets were independently analyzed. Information related to adjuvant therapy were available for 25 patients only (tamoxifen: N = 19; anastrozole: N = 5; no post-surgical therapy: N = 1). This study was conducted in accordance with the Declaration of Helsinki and approved by the Institutional Review Board of the “Regina Elena” National Cancer Institute of Rome and by the Leeds (East) Research Ethics Committee (06/Q1205/156). Written informed consent was not required as samples were anonymised to the research team^3^.

### Immunohistochemistry

TMAs were built from formalin-fixed paraffin-embedded (FFPE) material^3^. The immunohistochemical assessment of DDR markers was carried out in FFPE tissues using the following antibodies: anti-phospho-H2AX (Ser139) (clone JBW301) mouse monoclonal antibody (MAb) (Upstate) at the dilution of 1:500 (pH 8), anti-phospho-ATM (Ser1981) (clone 7C10D8) mouse MAb (Rockland) at the dilution of 1:200 (pH 6), anti-phospho-Chk2 (Thr68) (clone C13C1) rabbit MAb (Cell Signaling) at the dilution of 1:200 (pH 6), anti-phospho-ATR (Ser 428) (clone EPR2184) rabbit MAb (Abcam) at the dilution of 1:100 (pH 6), anti-phospho-Chk1 (Ser345) (clone 133D3) rabbit MAb (Cell Signaling) at the dilution of 1:150 (pH 6), anti-phospho-Wee1 (Ser642) (clone D47G5) rabbit MAb (Cell Signaling) at the dilution of 1:100 (pH 8), anti-phospho-RPA32 (Ser4/Ser8) rabbit polyclonal antibody (Bethyl) at the dilution of 1:100 (pH 6).

Positive and negative cases were classified consistently with the method exploited in our previous studies^23, 24^. DNA damage markers (γ-H2AX and pRPA32) were classified as high and low/negative using the median score of all tumors as the cut-off points, whereas DDR kinases were classified as positive when ≥10% of the neoplastic cells displayed a distinct nuclear immunoreactivity of any intensity. Hormone receptor immunoreactivity was scored using the Allred system and considered positive when >2^3^. Immunoreactivity was evaluated by two investigators (ADB and CE) blinded to baseline patient characteristics and treatment outcomes, and discordant cases were reviewed by a third observer (MM).

### Statistical analysis

The relationship between DDR markers was assessed with the Pearson’s Chi-squared test of independence (2-tailed) when they were analyzed as categorical variables. Correlations were assessed by Pearson’s correlation test for continuous variables (percentage of nuclear-expressing tumor cells). Overall survival (OS) was calculated as the time elapsing from diagnosis to death due to any cause. Five-, 10- and 15-year survival were calculated as the time from diagnosis to death due to any cause within a 5-, 10- and 15-year timeframe, respectively. Survival curves were generated by the Kaplan-Meier product-limit method and compared by log-rank test. Variables potentially affecting 10-year survival and OS were verified in uni- and multivariate Cox proportional hazard models. Statistical significance was set at p < 0.05. Statistical analyses were carried out using SPSS software (SPSS version 21, SPSS Inc., Chicago, IL, USA).

## Electronic supplementary material

Supplementary Figure 1 and supplementary table 1
