# Supplementary material for: Analysis of the ATR-Chk1 and ATM-Chk2 pathways in male breast cancer revealed the prognostic significance of ATR expression
Source: Sci Rep. 2017 Aug 14;7:8078. doi: 10.1038/s41598-017-07366-7 (PMC5556084; doi:10.1038/s41598-017-07366-7)
Supplement: Supplementary file 1 — Supplementary Figure 1 and supplementary table 1 [file 41598_2017_7366_MOESM1_ESM.pdf]

Title: Analysis of the ATR-Chk1 and ATM-Chk2 pathways in male breast cancer revealed the prognostic significance of ATR expression.

Authors: Anna Di Benedetto, Cristiana Ercolani, Marcella Mottolese, Francesca Sperati, Laura Pizzuti, Patrizia Vici, Irene Terrenato, Abeer M. Shaaban, Matthew P. Humphries, Luigi Di Lauro, Maddalena Barba, Ilio Vitale, Gennaro Ciliberto, Valerie Speirs, Ruggero De Maria, and Marcello Maugeri-Saccà

Supplementary Figure 1: Forest plot for subgroup analysis of 10-year survival by nodal status and Ki-67 levels.

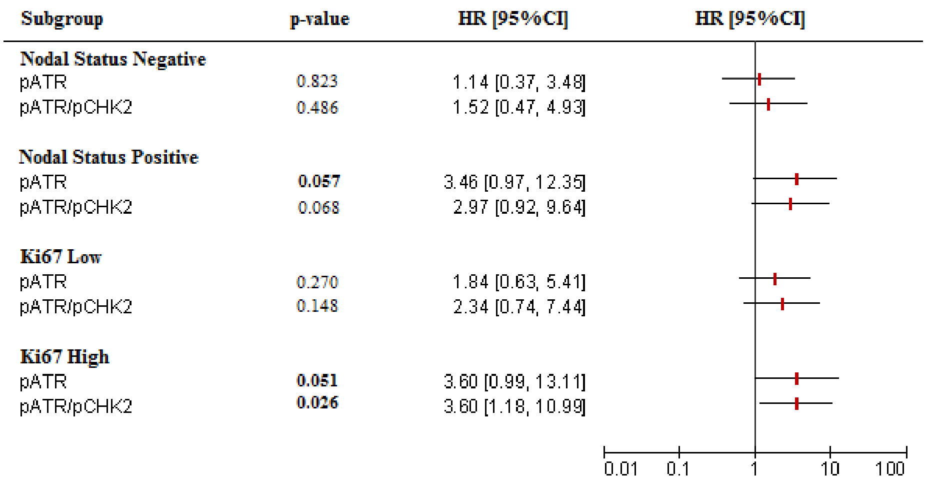

Supplementary Table 1: Correlations between DDR biomarkers in MBC patients.

**Pearson's Correlation Coefficient**	**pATR**	**pATM**	**pCHK2**	**pCHK1**	**pWEE1**	**pRPA32**	**γ-H2AX**
**pATR**	1						
**pATM**	0.30**	1					
**pCHK2**	-0.05#	0.11#	1				
**pCHK1**	0.15*	0.06#	-0.01#	1			
**pWEE1**	0.20**	0.31**	0.19**	0.13*	1		
**pRPA32**	0.60**	0.38**	-0.04#	0.21**	0.24**	1	
**γ-H2AX**	0.32**	0.22**	0.29**	0.07#	0.20**	0.30**	1

** p<0.01; *p<0.05; # not significant
